# Draft Genome Sequence of *Lactobacillus fermentum* NB-22

**DOI:** 10.1128/genomeA.00896-15

**Published:** 2015-08-13

**Authors:** A. V. Chaplin, A. N. Shkoporov, B. A. Efimov, A. P. Pikina, O. Y. Borisova, I. A. Gladko, E. A. Postnikova, A. E. Lordkipanidze, L. I. Kafarskaia

**Affiliations:** Pirogov Russian National Research Medical University, Moscow, Russian Federation

## Abstract

We announce here a draft genome sequence of *Lactobacillus fermentum* NB-22, a strain isolated from human vaginal microbiota. The assembled sequence consists of 190 contigs, joined into 137 scaffolds, and the total size is 2.01 Mb.

## GENOME ANNOUNCEMENT

*Lactobacillus fermentum* is a heterofermentative lactic acid bacterium (1) belonging to the phylum *Firmicutes*. It can be isolated from various sources, including mucosal microbiota of human and animals and fermented food. These bacteria are considered to possess probiotic properties, such as the absence of observed adverse effects related to their consumption (2), production of antimicrobial compounds (3), and immunomodulatory potential (4).

Multiple studies have explored the possibility of normalizing the vaginal microbiota using lactic acid bacteria. *L. fermentum* L23 was shown to produce both curative and protective effects in a murine model of vaginal tract infection caused by *Escherichia coli* (5). The combination of *L. fermentum* LF10 and *Lactobacillus acidophilus* LA02 was found to prevent recurrent vulvovaginal candidiasis in a small-scale clinical study without a control group (6). A large number of studies provided inconclusive but promising results of vaginal or oral administration of different combinations of *Lactobacillus* strains for the treatment of bacterial vaginosis and the prevention of its recurrence (7).

The molecular mechanisms of the putative probiotic activities of lactobacilli are largely unknown, and one of the ways to elucidate precise mechanisms and a diversity of host-microbe interactions is the collection and analysis of genomic data (8). This paper describes the draft genome sequence of *L. fermentum* NB-22, a strain that was isolated from human vaginal microbiota and was found to be perspective for usage as a probiotic due to its high adhesive and antagonistic properties.

Total culture DNA was sequenced using Illumina MiSeq. The reads were assembled *de novo* using CLC Genomics Workbench version 6.0.2. An assembly contained 190 contigs joined into 137 scaffolds using paired-end read data; the mean coverage was 114×. The total length of the sequence obtained was 2.01 Mb, showing a relatively small genome length in comparison with that of most other sequenced strains of *L. fermentum* (9–12); its genomic sequence was found to share 98.17% to 99.18% average nucleotide identity (ANI) with the other strains (13). The G+C content of the genome was 51.8%. An annotation made using PGAP (14) predicted 2,028 genes, including 1,938 protein-coding open reading frames. One of the small contigs (GenBank accession no. AYHA01000123) represented a unique 9-kb region without close homologs in complete and draft bacterial genomes; the genes within this locus encode the degradation of nitrogen-containing compounds, and one of the putative substrates might be allantoin.

### Nucleotide sequence accession numbers.

The *L. fermentum* NB-22 whole-genome shotgun project has been deposited at DDBJ/EMBL/GenBank under the accession no. AYHA00000000. The version described in this paper is version AYHA01000000.
